# The competing effects of microbially derived polymeric and low molecular-weight substances on the dispersibility of CeO_2_ nanoparticles

**DOI:** 10.1038/s41598-018-21976-9

**Published:** 2018-02-26

**Authors:** Yuriko Nakano, Asumi Ochiai, Keisuke Kawamoto, Ayaka Takeda, Kenta Ichiyoshi, Toshihiko Ohnuki, Michael F. Hochella, Satoshi Utsunomiya

**Affiliations:** 1 Department of Chemistry, Kyushu University, 744 Motooka, Nishi-ku, Fukuoka-shi 819-0395 Japan; 2 Laboratory for Advanced Nuclear Energy, Institute of Innovative Research, Tokyo Institute of Tecnology, 2-12-1 Ookayama, Meguro-ku, Tokyo 152-8550 Japan; 3 Department of Geosciences, Virginia Tech, Blacksburg, VA 24061 USA; 4 Subsurface Science and Technology Group, Energy and Environment Directorate, Pacific Northwest National Laboratory, Richland, WA 99352 USA

## Abstract

To understand the competing effects of the components in extracellular substances (ES), polymeric substances (PS) and low-molecular-weight small substances (SS) <1 kDa derived from microorganisms, on the colloidal stability of cerium dioxide nanoparticles (CeNPs), we investigated their adsorption to sparingly soluble CeNPs at room temperature at pH 6.0. The ES was extracted from the fungus *S. cerevisiae*. The polypeptides and phosphates in all components preferentially adsorbed onto the CeNPs. The zeta potentials of ES + CeNPs, PS + CeNPs, and SS + CeNPs overlapped on the plot of PS itself, indicating the surface charge of the polymeric substances controls the zeta potentials. The sizes of the CeNP aggregates, 100–1300 nm, were constrained by the zeta potentials. The steric barrier derived from the polymers, even in SS, enhanced the CeNP dispersibility at pH 1.5–10. Consequently, the PS and SS had similar effects on modifying the CeNP surfaces. The adsorption of ES, which contains PS + SS, can suppress the aggregation of CeNPs over a wider pH range than that for PS only. The present study addresses the non-negligible effects of small-sized molecules derived from microbial activity on the migration of CeNP in aquatic environments, especially where bacterial consortia prevail.

## Introduction

The cerium dioxide (CeO_2_, $F{\rm{m}}\bar{3}m$) nanoparticle (CeNP) is a nanomaterial that is finding a wide variety of applications to a vast number of products involving fuel additives^1^, fuel cell components^2^, biomedical applications^3,4^, combustion accelerators and abrasives^5,6^, and specialized polishing agents^7^. With all of these applications, it is inevitable that CeNPs will be found in the environment. Unfortunately, *in vitro* and *in vivo* experiments with CeNPs have shown that this material can cause chronic toxicity to aquatic organisms^8^, cell death to *E. coli*^9^, increase of reactive oxygen species levels relevant to human lung cells^10^, and decrease of glutathione levels in cultured human lung epithelial cells^11^. Due to their small size, ~10 nm, the inhaled CeNPs can penetrate into the deep respiratory system^12^ and potentially cause adverse health effects despite the existing study reported that CeNPs have low human toxicity^13^. Thus, the distribution and migration behavior of CeNP, as well as other engineered nanoparticles in the ambient environment, is a central issue that requires careful monitoring and modeling^14^. The mobility of CeNP follows the general rules for colloid transport in surface and subsurface environments^15–19^. Colloid transport can be controlled by several processes: sedimentation, filtering effects, hydrodynamic chromatographic effects, and capillary effects. All of these processes are largely dependent on the aggregation processes of CeNPs in natural aquifers^17,20^.

The aggregation of colloids is mainly constrained by several factors, including solution pH, electrolyte concentrations^21–23^, adsorbed ions^23,24^, and adsorbed organic matter^23,25^. On the other hand, natural and engineered nanoparticles, including CeNPs, can encounter microbial consortia in the subsurface environment^26^ due to the ubiquitous occurrence of microorganisms^27,28^. During the interaction between microorganisms and nanoparticles, the extracellular substances (ES) that are released by the microorganisms^29,30^, as essential constituents to form biofilms^31^, adsorb onto the nanoparticle’s surface and occasionally lead to particle dissolution^32^, promoting electron transfer^33^, and changing the dispersibility of the nanoparticles in solution^29^. The polymer substances (PS) included in the ES category are generally composed of 40–95% polysaccharides, <1–60% protein, <1–10% nucleic acids, and <1–40% lipids^34,35^. Adsorption of the PS onto the nanoparticles changes the zeta (ζ) potential of aggregates and promotes the dispersibility of particles, increasing the critical aggregation concentration^29,36–39^. Adeleye *et al*.^36^ reported that extracellular PS adsorbed onto Cu and CuO nanoparticles can change the ζ potential from positive to negative at pH 4 and narrowed the ζ potential range. Miao *et al*.^39^ also reported the enhanced stability of CuO nanoparticles after adsorption of extracellular PS and polysaccharides due to the electrostatic repulsion and formation of a steric barrier. Adeleye and Keller^37^ carried out adsorption experiments of extracellular PS onto TiO_2_ nanoparticles. This resulted in a reversal of the surface charge and enhanced particle stabilization. Lin *et al*.^38^ performed adsorption experiments for extracellular PS onto TiO_2_ nanoparticles and found that both electric repulsion and steric hindrance were mechanisms of stabilization with increasing mass of the adsorbed extracellular PS. Our previous study^29^ revealed enhanced stabilization of CeNPs through steric hindrance and the critical aggregation concentration of NaCl increased from 10 mM to 250 mM when ES was adsorbed onto CeNPs. Despite the fact that effects of extracellular PS have been explored, the previous studies have focused on the polymers only, excluding the effect of small molecules in the ES. Thus, there is limited knowledge on the total competing effects of ES components including small substances. The aim of the present study is to understand the properties of the PS in microbially derived ES, PS, and SS. Secondarily, we aim to evaluate their competing effects on the adsorption processes onto CeNPs, changes in the CeNP surface properties, and the aggregation and sedimentation of CeNPs at various pHs.

## Materials and Methods

### CeO_2_ nanoparticles (CeNPs)

Synthetic CeNPs were commercial products purchased from Strem Chemicals, Inc., Newburyport, MA, USA, (part# 58–1400, ~7 nm). The CeNPs had spherical shape and the average diameter was ~7 nm. The surface area was determined to be 70.2 m^2^ g^−1^ using a BET single point analysis, which was smaller than that calculated assuming fully dispersed spherical nanoparticles having <10 nm in size. This indicates that the CeNPs already aggregated prior to use in the present experiments. For a detailed description of these CeNPs, see references^29,30^.

### Preparation of the extracellular substances (ES), the extracellular polymeric substances (PS) and the extracellular small substances (SS)

In the present study, *Saccharomyces cerevisiae* (X-2180) was used as a representative microorganism. First, *S. cerevisiae* was harvested in 200 mL of sterilized YPD medium, which was composed of 10 g L^−1^ yeast extract, 20 g L^−1^ peptone, and 20 g L^−1^ dextrose. The yeast was incubated for 20 h on a rotary shaker at 120 revolutions per minute (rpm) at 25 ± 1 °C. The suspension of the yeast cells were centrifuged for 10 min at 3000 rpm to be separated. The separated cells were washed three times with 1 mmol L^−1^ NaCl solution. The yeast cells were put in a polypropylene tube filled with 50 mL of 1 mmol L^−1^ NaCl. In all solutions the cell density was adjusted to 2.0 ± 0.1 dry g L^−1^. The pH of the solutions was initially adjusted to 3.0 ± 0.1 with 1.0 mol L^−1^ HNO_3_ solution. In our previous study^29^, a high concentration of organic matter was extracted at this pH and the composition was similar to the organic matter extracted at higher pHs. A pH meter (TOA tpx-999i; PCE108CW-SR) equipped with an electrode was used to measure pH.

After extracting ES for 72 h, the suspension was filtered through a polytetrafluoroethylene (PTFE) membrane filter (Advantec) with 0.20 μm pore size to remove the yeast cells. The filtrate was named as the ES solution. This ES solution contained both polymers and low-molecular-weight species. A portion of ES was dialyzed for 72 hours at 4 °C using a 1000 MWCO Spectra/Por® 7 (Spectrum) cellulose dialytic membrane. The volume ratio of the ES to ultrapure water was set to 1:3. The water outside the dialytic membrane was exchanged with ultrapure water every 24 hours. This outside solution after the first 24 hours of dialysis was labeled the “extracellular small substances (SS)” solution. The conductivity after 72 hours was measured to be ~0.0 μS. The solution that remained in the membrane tube was labeled PS. The ES, PS, and SS solutions were preserved at 4 °C in a refrigerator and the solutions were adjusted back to room temperature prior to use in experiments.

The morphology of the ES was observed by scanning probe microscopy (SPM, DimensionIcon, Bruker AXS, Billerica, USA). The observations were performed under ambient atmospheres using a ScanAsyst probe (ScanAsyst-Air). The specimen for SPM was prepared by dropping the ES solution onto the cleaved pristine surface of biotite and air-dried. Then, the specimen was rinsed with ultrapure water three times and air-dried again.

The ES contained various kinds of polymers, organic matter, and inorganic ions, such as H_3_PO_4_. The total phosphate concentrations in the ES, PS, and SS were determined using inductively coupled plasma atomic emission spectrometry (ICP-AES; Agilent 7500c). The detection limit of P was 15 ppb. The concentrations of dissolved organic carbon (DOC) were determined by using a total organic carbon analyzer (TOC; TOC-VE, Shimadzu). The detection limit was 50 μg L^−1^ and the error was <2%. To further characterize the ES, the dried ES, PS, and SS were analyzed using an attenuated total reflectance Fourier transform infrared spectrometer (ATR-FTIR; Jasco, FT/IR-620) equipped with a deuterated L-alanine triglycine sulfate (DLATGS) detector, a single bounce attenuated total reflectance attachment, and a ZnSe crystal. Thirty-two spectra were obtained with a spectral resolution of 4 cm^−1^ and averaged. To prepare the dried samples, the pH of the ES solutions was first adjusted to 6.0 ± 0.1 with 1.0 mol L^−1^ NaOH solution. The ES, PS, and SS solutions were lyophilized and preserved at −10 °C until the measurement. In addition to the FTIR analysis, elemental analysis was completed on the lyophilized ES, PS, and SS to determine the concentrations of C, N, and H.

### Adsorption of ES, PS, and SS onto the CeNPs

The 5000 mg L^−1^ CeNPs stock suspension was prepared and ultra-sonicated for 10 min. Five different solutions were prepared in the present experiment: (i) 1 mM NaCl (control); (ii) 1 mM NaCl + 160 μM H_3_PO_4_ (160 μM P), of which the P concentration was adjusted to that of the ES solution in the previous study^29^; (iii) ES solution containing1 mM NaCl solution (conditions during the extraction procedure); (iv) PS + 1 mM NaCl, to adjust the ionic strength to be similar to the other solutions; and (v) SS + 1 mM NaCl. The pH of these suspensions was adjusted to 6.0 ± 0.1 with NaOH. Each of these five solutions were mixed with an aliquot of CeNPs stock solution, in which the concentration of CeNPs was set to 100 mg L^−1^ so that multiple analytical techniques could be employed. In this study, we did not adjust the C content prior to the adsorption experiments, because the C content does not reflect the actual concentration of specific organic molecules. All ES, PS, and SS contain organic matter with various molecular weights. Thus, it is difficult to quantify the actual concentrations of the non-specified molecules. Rather, the CeNP surfaces were saturated with the organic matters that have concentrations as prepared in the experiments.

High-Angle Annular Dark-Field Scanning TEM (HAADF-STEM) and energy-dispersive x-ray spectroscopy (EDX) were completed using a scanning transmission electron microscope (STEM, JEOL, JEM-ARM200CF and JEM-ARM200F, Akishima, Japan). The TEM specimens were prepared by desalinating three times with ultrapure water and dropping the suspension sample on a 300 mesh Cu with Ge or holey carbon supporting membrane followed by air-drying. For the ATR-FTIR analyses, ES, PS, or SS were adsorbed onto the CeNPs at pH 6.0. These suspensions were statically reacted for 24 h. The duration of 24 hours is enough to achieve the apparent equilibrium in this experiment according to our previous study^29^. After the adsorption, the CeNPs associated with organic matter were separated using a 0.025 μm nitrocellulose membrane filter and lyophilized. The analytical procedure of ATR-FTIR for ES, PS, or SS adsorbed to CeNPs was the same as the one described in the previous section.

A Zeta Sizer Nano ZEN (Malvern Instruments Inc) was used to measure the ζ potential and average hydrodynamic diameter for the CeNPs suspensions in 1 mM NaCl solution with a capillary cell. The starting pH was set to 6.0 and the pH was shifted to the targeted value using NaOH or HNO_3_.

## Results and Discussion

### Characterization of ES, PS, and SS

The composition of the ES extracted in 1.0 mmol L^−1^ NaCl after 72 hours of incubation is summarized in Table 1. The ES contains ~172 mg L^−1^ organic carbon, ~0.44 mmol L^−1^ K^+^, and ~0.59 mmol L^−1^ total P. The compositions of PS and SS are also given in Table 1. The ES used in the present study contained 2–4 times higher concentrations of organic and inorganic species than the ES characterized in our previous study^29^. The amounts of dissolved organic species in the solutions before and after the adsorption experiments are given in Table S1.

**Table 1:** Major composition of ES solution extracted from *S. cerevisiae* in 1.0 mmol L^−1^ NaCl for 72 hours of incubation.

Compound	Concentration			
	ES	PS	SS	ES^29^
Dissolved Organic Carbon (mg L^−2^)	172 ± 7.4	23.3 ± 0.1	36.1 ± 0.3	72.3 ± 19.5
Dissolved Nitrogen (mg L^−1^)	84.2 ± 3.7	9.67 ± 0.02	20.1 ± 0.1	41.5 ± 11.2
Total phosphate (mmol L^−1^)	0.59 ± 0.003	0.090 ± 0.001	0.107 ± 0.001	0.157 ± 0.07
K^+^ (mmol L^−1^)	0.44 ± 0.002	0.016 ± 3E-04	0.108 ± 0.001	0.489 ± 0.03

PS stands for the polymer substances >1 kDa contained in ES. SS stands for the small substances <1 kDa.

**Figure 1:**
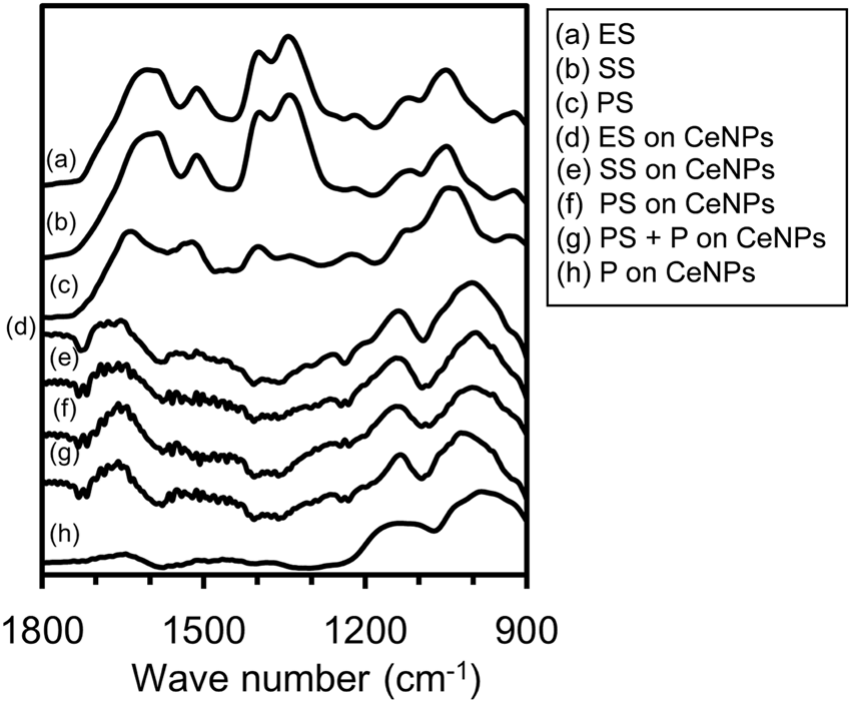
ATR-FTIR spectra of the lyophilized samples: (**a**) ES, (**b**) SS, (**c**) PS, (**d**) CeNP reacted with ES in 1.0 mM NaCl at pH 6.0 for 24 hours, (**e**) CeNP reacted with SS in 1.0 mM NaCl at pH 6.0 for 24 hours, (**f**) CeNP reacted with PS in 1.0 mM NaCl at pH 6.0 for 24 hours, (**g**) CeNP reacted with PS + 160 µM P in 1.0 mM NaCl at pH 6.0 for 24 hours, and (**h**) CeNP reacted with 160 µM of P in 1.0 mM NaCl at pH 6.0 for 24 hours. Spectra (**d**–**h**) are difference spectra (pristine CeNP spectrum was subtracted from the original spectrum).

Figure 1 shows ATR-FTIR spectra (900 to 1800 cm^−1^) of the lyophilized samples before the adsorption experiments: ES (a), SS (b), and PS (c). The peak assignments, with references, are summarized in Table 2. In line (a), the absorption band at ~1397 cm^−1^ is assigned to symmetric stretching of COO^−^ groups (ν_s_ C–O) that are included in proteins and polypeptides, and carboxylated polysaccharides^40–42^. The band at ~1604 cm^−1^ is assigned to the stretching vibration of C=O groups derived from amide I bonds, which represent amides associated with proteins and polypeptides. The band at ~1518 cm^−1^ is assigned to the stretching vibration of C–N groups and deformation vibration of N–H groups included in amide II bonds, which correspond to –CO–NH– of proteins and polypeptides^41–43^. Thus, the two bands at 1604 and 1513 cm^−1^ suggest that the ES contains proteins and polypeptides. The band at ~1119 cm^−1^ can be assigned to ring vibrations of C–O–C bonds included in polysaccharides and the stretching vibrations of P=O bonds in proton-dissociated orthophosphate. The band at ~1343 cm^−1^ is assigned to the carbon backbone coupled with C-O and P-O stretching^44^. Further, the band at ~1050 cm^−1^ is assigned to symmetric stretching vibrations of P=O derived from phosphoryl groups^24,40,45^. It is difficult to separate the phosphate bands and the polysaccharide vibration bands due to their overlaps; however, the ES released from *S. cerevisiae* typically contains both polysaccharides and phosphoryl species, which are also included in the ES released from *Bacillus subtilis*^40,46^ and *Pseudomonas aeruginosa*^45,47^.

**Table 2:** Band assignment in the FTIR spectra based on the previous studies.

Measurement data k (cm^−1^)			Literature data k (cm^−1^)	Band assignment
ES	PS	SS		
1604	1640	1586	1660^a^	*v*C=O of amides associated with proteins (amide I)
1513	1528	1513	1544^a^	*δ*N-H and *v*C-N in -CO-NH- of proteins (amide II)
			1449^a^	*δ*sCH_2_, and *δ*C-OH
1397	1393	1396	1403^a^	*v*_s_C-O of COO- groups
1343	1340	1341	1330^c^	Carbon backbone coupled with C-O and P-O stretching
1221	1237	1221	1242^a^	*v*_sa_P=O of phosphodiester backbone of nucleic acid; may also be due to phosphorylated proteins
1119		1116	1127^a^	O-H deformation, *v*C-O, ringvibrations of polysaccharides
1050	1043	1049	1075^a^	*v*P=O of H_2_PO_4_^−^
			1078^b^	*v*_s_P=O of phosphodiester backbone of nucleic acid, phosphomonoester, phosphorylated proteins, and C-OH stretch
924		925	920^a^	Asymmetric ester O-P-O stretching modes from nucleic acids

^a^Omoike and Chorover (2004 and 2006)^40,45^. ^b^Tejedor-Tejedor and Anderson (1990)^24^. ^c^Sheals *et al*. (2002)^44^.

The FTIR spectrum of SS (b), molecules smaller than 1 kDa, is similar to that of the ES before dialysis. Although the peak position of the PS spectrum is similar to that of the ES spectrum, there is a slight difference in the relative intensity between the peaks. The relative intensity of the peaks derived from phosphate (1044 cm^−1^) and carboxyl (1399 cm^−1^) groups was weaker than that of proteins (1521 cm^−1^ and 1635 cm^−1^) in the PS fraction. These results indicate that SS contains almost the same compounds as ES, such as inorganic phosphate, amino acids, polysaccharides, and polypeptide, whereas PS contains mainly polysaccharides, proteins and polypeptides of larger molecular sizes >1 kDa.

### Morphology of polymeric substances within the ES

**Figure 2:**
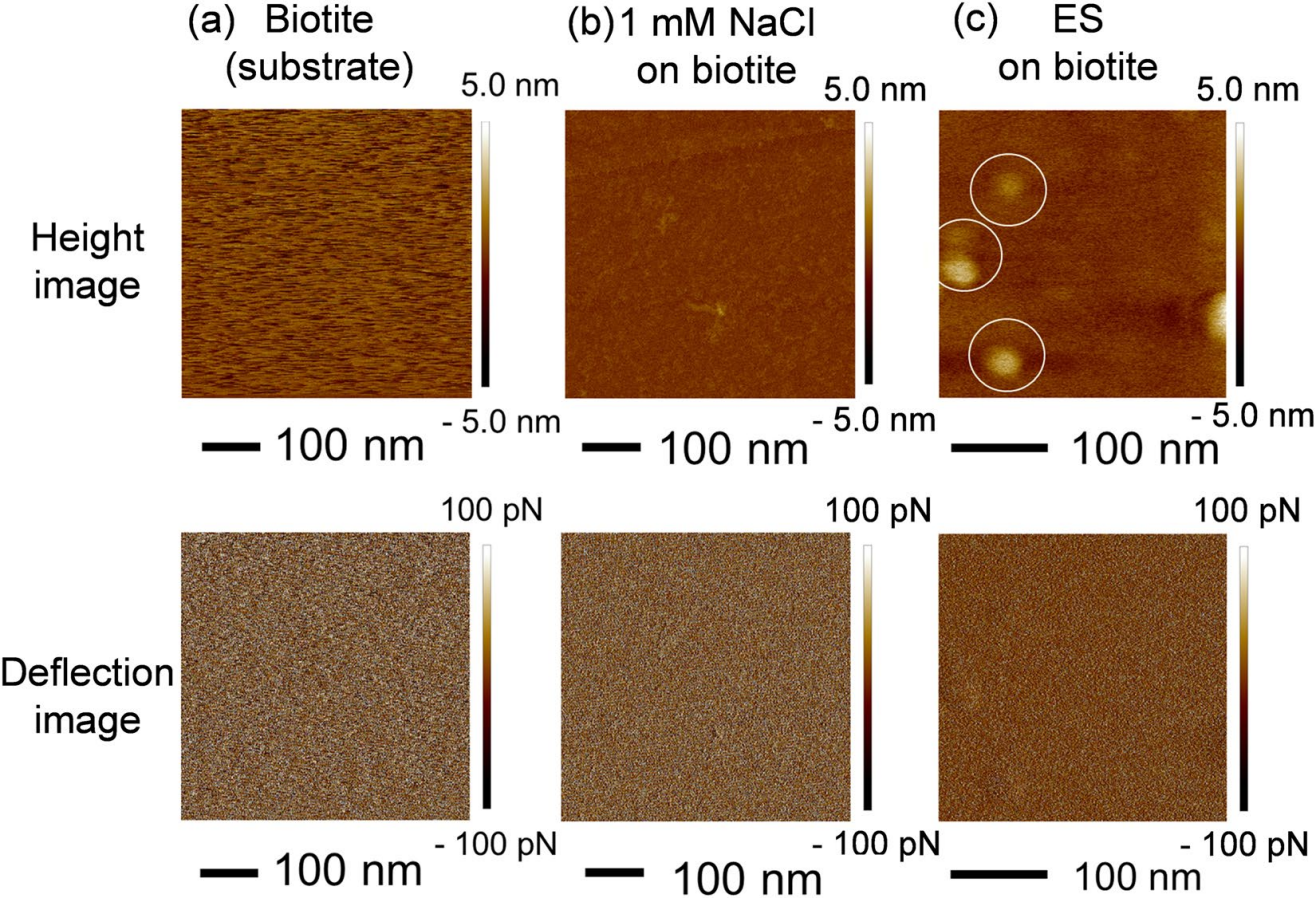
(Top panels) Height images recorded with AFM. (Bottom panels) Deflection images by AFM. (**a**) Fresh surface of cleaved biotite. (**b**) Surface of the cleaved biotite recorded after contact with 1 mM NaCl and washing with ultrapure water thrice. (**c**) Surface of the cleaved biotite recorded after contact with ES solution and washing with ultrapure water thrice.

The topological and phase contrast AFM images were obtained for the freshly cleaved biotite (Fig. 2a), the freshly cleaved biotite without the ES after desalination (Fig. 2b), and the ES adsorbed onto the freshly cleaved biotite after desalination (Fig. 2c), which showed the presence of nanoparticles 20–30 nm in diameter and ~3 nm in height (Fig. 2c). The same mode images without ES after desalination did not show any nanoparticles on the surface of cleaved biotite (Fig. 2b). Thus, the particles detected in the former images (Fig. 2c) are polymeric substances of the ES. The shallow height of the nanoparticles indicates a flattened shape after substrate adhesion; thus, the true particle size in solution is likely smaller than 20 nm.

### STEM of the ES + CeNPs, PS + CeNPs, and SS + CeNPs

**Figure 3:**
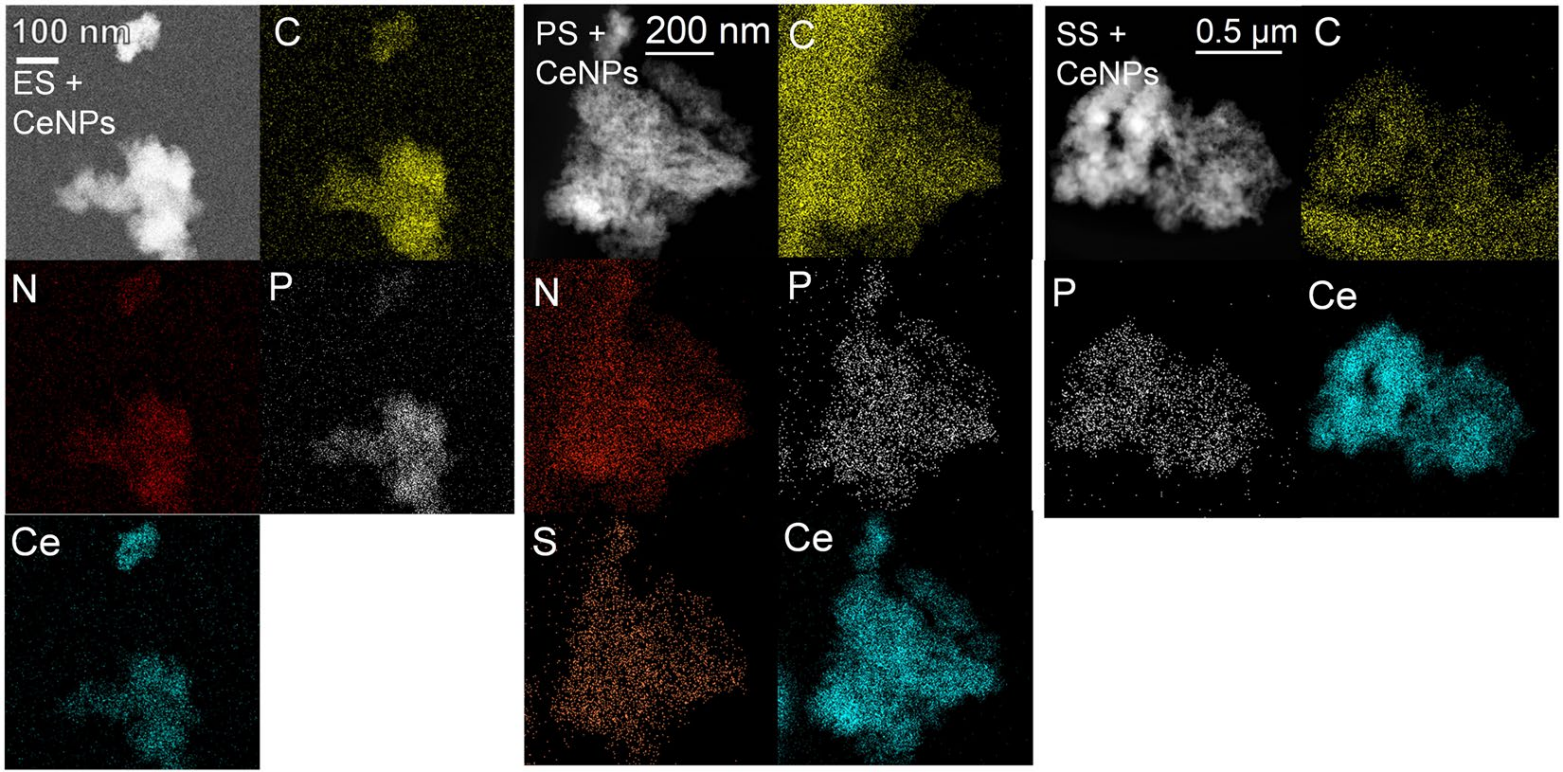
HAADF-STEM image with the elemental maps of the CeNP specimen after contact with ES, SS, or PS at pH 6.0 in 1.0 mM NaCl for 24 hours followed by desalination washing with ultrapure water thrice. The suspension of ES + CeNPs was dispersed on the Ge-mesh without using C, while the samples of PS + CeNPs and SS + CeNPs were prepared on holey carbon mesh with a Cu supporting grid.

**Figure 4:**
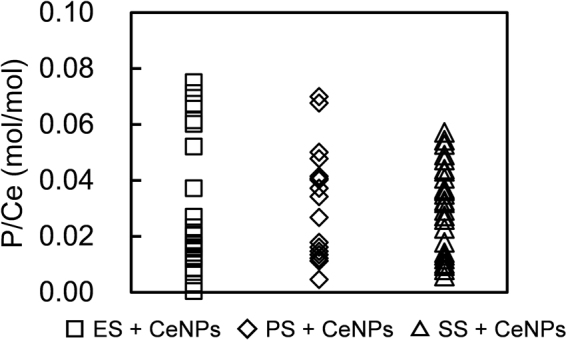
P/Ce molar ratio calculated from the EDX spectra: ES + CeNPs (square), PS + CeNPs (diamond), and SS + CeNPs (triangle).

Figure 3 shows that HAADF-STEM image and the EDX elemental maps of the samples. ES + CeNPs, PS + CeNPs, and SS + CeNPs exhibit CeNP aggregation ranging from 100 to 500 nm, on which C, N, and P are distributed uniformly, indicating that the polymeric substances of the ES adsorbed onto the CeNP surface. Note that the C map contains interference from the holey carbon supporting mesh. In the PS + CeNPs specimen, the EDX spectrum reveals a clear peak of the S K-line in an aggregate, derived from thiol groups, which is likely attributed to the presence of amino acids such as cysteine and methionine. The P/Ce molar ratio on the aggregates in ES + CeNPs, PS + CeNPs, and SS + CeNPs varies between 0.01 to 0.08, indicating that the adsorption of ES, PS, and SS to CeNP is not homogeneous (Fig. 4). Representative EDX spectra for ES + CeNPs, PS + CeNPs, and SS + CeNPs can be found in Fig. S1.

### FTIR of ES, PS, and SS adsorbed to CeNPs

ATR-FTIR difference spectra of the experimental samples are shown in Fig. 1: ES + CeNPs (d), SS + CeNPs (e), PS + CeNPs (f), PS + 160 μM P + CeNPs (g), and 160 μM P + CeNPs (h). The CeNPs spectrum was subtracted from the raw spectra to display only the spectra of the adsorbed species. All of the spectra after the adsorption treatment appear similar. After adsorption, the bands for phosphate were broadened, indicating the formation of inner-sphere complexes on the CeNPs, as previously reported^29^, and proteins and polypeptides adsorbed preferentially onto the CeNP surfaces. Interactions between the extracellular PS and metal oxides generally occur via amide, hydroxyl, and carboxylic groups on the PS amino acids in addition to the phosphate groups from phospholipids or nucleic acids^37^. The amide I peak, derived from proteins and polypeptides, shifted toward higher frequencies and the peak derived from the carboxyl group became minimized after adsorption to the CeNPs, regardless of dialysis treatment. The predominant adsorption of proteins from the bacterial extracellular PS to metal oxides has also been reported in several previous studies^40,48^ by forming inner-sphere complexes^45^. Proteins can adsorb onto hydrophilic surfaces and the shift in the peak position typically occurs due to protein conformational changes after adsorption^49–52^.

Although the chemical compositions between ES, PS, and SS were different, the FTIR spectra after adsorption to CeNPs appear identical, strongly suggesting that the molecules adsorbing onto the CeNP surfaces possess similar functional groups, despite the molecular size differences in the ES, PS, and SS constituents.

### The effects of ES, PS, and SS on the surface electric potential (ζ potential)

**Figure 5:**
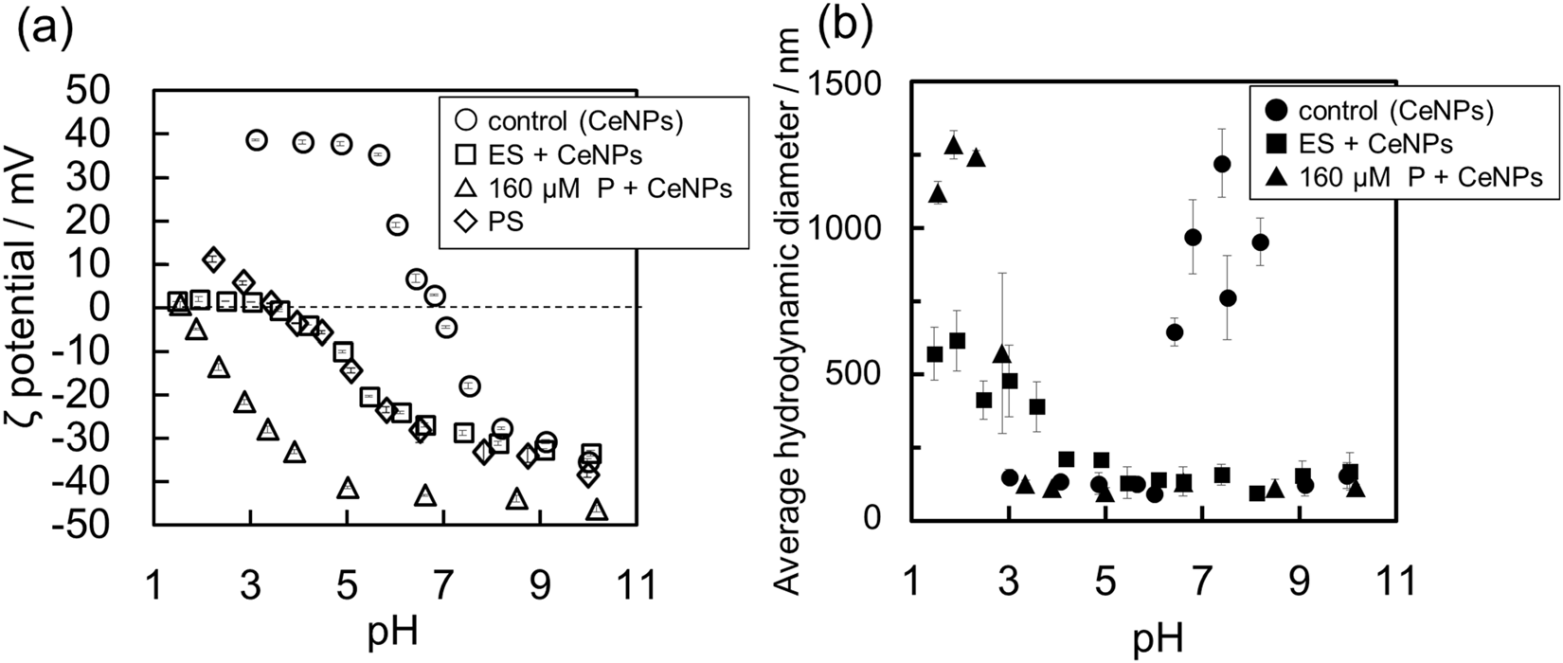
(**a**) ζ potentials of CsNPs as a function of pH after the adsorption experiment in 1.0 mM NaCl at pH 6.0 ± 0.1 after 20–24 hours under various conditions: control (open circle), ES + CeNPs (open square), 160 μM P + CeNPs (open triangle), and PS only (open diamond). (**b**) Average hydrodynamic diameter of CeNPs aggregates as a function of pH after the same adsorption experiments: control (closed circle), with ES (closed square), and with 160 μM P (closed triangle).

Figure 5a shows the pH dependence of ζ potential for: CeNPs (control), ES + CeNPs, 160 μM P + CeNPs, and PS only. The point of zero charge (PZC) of CeNPs (control) was determined to be 6.9, which was within the range of the reported PZC values from several literature sources; 6.5–8.0^21,53^. The ζ potentials of the CeNPs in 160 μM P were −40 to −50 mV at pH > 5.0, and the isoelectric point (iep) was determined to be ~1.6. In the pH 6 solution, the ζ potential decreased as H_2_PO_4_^−^ and HPO_4_^2−^ adsorbed to the CeNPs. In the ES + CeNPs, the ζ potentials were plotted between the control and 160 μM P values. The P concentration (592 μM) in the present ES was determined to be higher than that measured in our previous study^29^, but within the same order of magnitude. As reported in^29^, phosphate in the ES also adsorbed to CeNPs, although the ζ potential was not affected by the adsorbed phosphate. Furthermore, the ζ potentials of PS were plotted almost identical to the plots of ES + CeNPs. The ζ potentials of ES (Fig. S2) were plotted deviated from PS and ES + CeNPs, indicating that the ES compounds adsorbed onto the CeNP is similar to PS rather than the total ES compounds. It was impossible to measure the ζ potential of SS due to their small sizes. The electrophoretic mobility distribution for the ES + CeNPs was similar to that for PS at pH 2.9–10.0 (Fig. S3). In the diagram for PS at pH 9.99 (Fig. S3), the single peak split into multiple peaks at high pH, most likely because there were several aggregates with different functional groups on the CeNP surface. In the case of ES + CeNPs, the peaks were located at the same mobility value  as the case of PS, which may indicate that the large molecules with similar specific functional groups preferentially adsorbed onto the CeNP surfaces. The effect of phosphate adsorption did not appear in the ζ potential in the ES solution because the preferential adsorption of macromolecules, such as proteins and polypeptides, on the outermost surface hinders the effects of orthophosphate.

**Figure 6:**
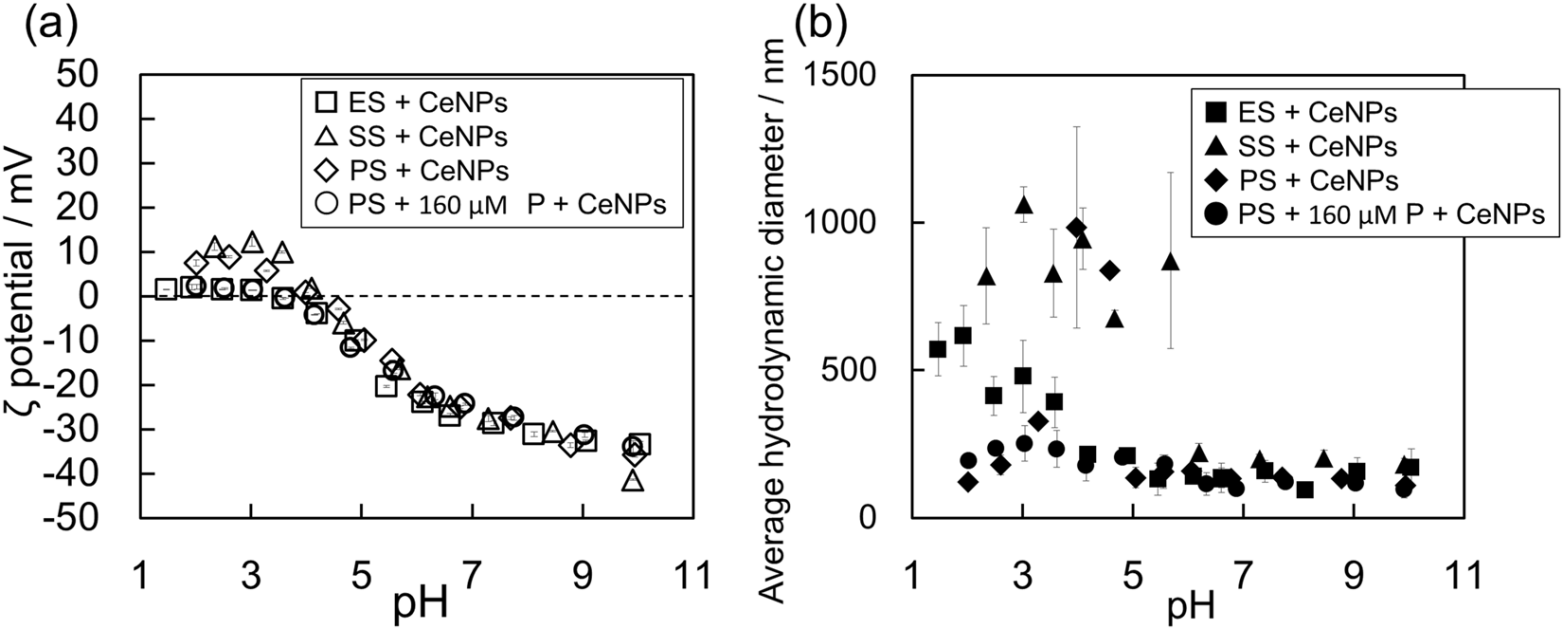
(**a**) ζ potentials of CsNPs as a function of pH after the adsorption experiment in 1.0 mM NaCl at pH 6. 0 ± 0.1 after 20–24 hours under various conditions: with ES (open square), with SS (open triangle), with PS (open diamond), and with PS + 160 μM P (open circle). (**b**) Average hydrodynamic diameter of CeNP aggregates as a function of pH after the same adsorption experiments: with ES (closed square), with SS (closed triangle), with PS (closed diamond), and with PS + 160 μM P (closed circle).

**Figure 7:**
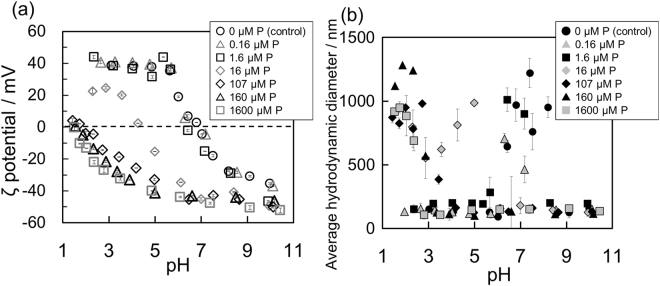
(**a**) ζ potentials of CsNPs as a function of pH after the adsorption experiment in 1.0 mM NaCl at pH of 6.0 ± 0.1 after 20–24 hours under various conditions: control (open black circle), with 0.16 µM P (open gray triangle), with 1.6 µM P (open black square), with 16 µM P (open gray diamond), with 107 μM P (open black diamond), with 160 μM P (open black triangle), and with 1600 μM P (open gray triangle). (**b**) Average hydrodynamic diameter of CeNP aggregates as a function of pH after the same adsorption experiments: control (closed black circle), with 0.16 µM P (closed gray triangle), with 1.6 µM P (closed black square), with 16 µM P (closed gray diamond), with 107 μM P (closed black diamond), with 160 μM P (closed black triangle), and with 1600 μM P (closed gray triangle).

The ζ potentials of three additional systems (PS + CeNPs, PS + 160 μM P + CeNPs, and SS + CeNPs) were also plotted in Fig. 6a, confirming that the presence of inorganic phosphate in the ES did not influence the ζ potential of CeNP in any system where PS was present; that is, the ζ potential of CeNPs reacted with ES was governed by the polymers in the ES rather than the small charged molecules, such as phosphate. In addition, the ζ potential of SS + CeNPs, which contained inorganic phosphate, also exhibit the same trend as that of ES + CeNPs. There are two factors that caused the similarity. One factor is the steric barrier created by the organic matter, even by molecules size smaller than 1 kDa, because the FTIR spectra of ES, PS, and SS revealed that the functional groups of the compounds adsorbed on the CeNPs were nearly identical (Fig. 1). The other factor is the decreased concentration of SS due to dialysis. The SS solution was diluted to approximately one quarter, implying the possibility of decreased amounts of adsorbed inorganic phosphate in the ES. Indeed, the adsorption experiment of inorganic phosphate using various concentrations of phosphate revealed that the ζ potential increases gradually as the P concentration decreases, and the pH dependence at the P concentration of 1.6 µM, which is two orders of magnitude less than the P concentration in ES, became identical to that of the control (Fig. 7a). In addition, the average hydrodynamic diameter also changed concurrently; when P concentration decreased from 160 μM to 16 μM, the ζ potential shifted to a positive value, the iep shifted from ~1.6 to ~4.3, and the pH, at which the average hydrodynamic diameter becomes the maximum, shifted from ~1.9 to ~5.0. The pH dependence of the average hydrodynamic dimeter of the SS + CeNPs appeared similar to that of P + CeNPs (16 µM) (Fig. 7b). When the CeNPs were exposed to 107 µM P, the same P concentration in SS, the iep shifted from ~1.6 to ~2.0, though the pH dependence of the ζ potential was almost identical to the 160 µM P case. Thus, the pH dependence of the ζ potentials for SS + CeNPs and ES + CeNPs can be ascribed to the similar characteristics of the polymeric substances, even when of different molecular sizes.

### The effects of ES, PS, and SS on the size of the aggregates

Figure 5b shows the average hydrodynamic diameter of CeNPs in three conditions: (i) 1.0 mM NaCl; (ii) 1.0 mM NaCl + 160 μM H_3_PO_4_; (iii) ES + 1 mM NaCl, over pH 1–11. The size increases at near the iep under all conditions. This indicates that the electrostatic repulsive force resulted from the outermost charge of the particle constrains the aggregation behavior of CeNPs.

When the pH was less than 3, the ζ potentials of the ES + CeNPs and the 160 μM P + CeNPs cases were both close to zero, meaning that the electrostatic repulsive force was not effective under low pH conditions (Fig. 5a). However, the size of ES + CeNPs was less than half of that of 160 μM P + CeNPs, indicating that the steric barrier formed by the polymeric substances effectively suppressed the aggregation of CeNPs. Indeed, as described above, the FTIR results indicated a preferential adsorption of proteins onto the CeNP surfaces, causing steric hindrance^39^. On the other hand, between pH 3 and 4, the average particle size of ES + CeNPs was larger than that of the 160 μM P + CeNPs and control cases. Under this pH condition, the ζ potential of ES + CeNPs was close to zero, while that of 160 μM P + CeNPs was as low as −30 mV. This indicates that the aggregation behavior of CeNPs was constrained by both electrostatic and steric repulsion, and the effects of electrostatic repulsion were greater than that of the steric barrier.

Figure 6b shows the pH dependence of the average hydrodynamic diameter of ES+ CeNPs, SS + CeNPs, PS + CeNPs, and PS + 160 μM P + CeNPs. The average hydrodynamic diameter for SS + CeNPs exhibited a different trend from that of 160 μM P + CeNPs or ES + CeNPs, most likely because the electrostatic repulsive forces in SS + CeNPs were less than that of the 160 μM P + CeNPs case, due to the lower P concentration in SS, and the steric barrier derived from the molecules <1 kDa in SS was weaker than that in the ES + CeNPs case. In the PS + CeNPs case, the average hydrodynamic diameter increased around the iep of PS itself, whereas that in the PS + 160 μM P + CeNPs case showed only a slight increase around pH ~3. The difference might be attributed to the presence of P adsorbed onto CeNPs rather than PS adsorption on to the CeNPs. CeNP aggregation should have been promoted at between pH 3 to 5, near the iep of PS (~3.5), because the ζ potential is determined by the largest molecules, regardless of the presence of phosphate. However, aggregation appeared to be suppressed by the inorganic phosphate adsorbed onto CeNPs, likely because the repulsive forces derived from the adsorbed inorganic phosphate became predominant when the distance between CeNP surfaces become sufficiently small. At pH <3, which is close to iep (pH ~1.6) of 160 μM P + CeNPs, both electrostatic repulsive forces and steric barriers from the adsorbed PS reduced aggregation. Although the same trend can be seen in ES + CeNPs, aggregation in PS + 160 μM P + CeNPs was suppressed more profoundly than that in ES + CeNPs. The ES contains free cations and small organic molecules, including amino acids with carboxyl groups. Thus, in the ES + CeNPs system, the electric repulsive forces of inorganic phosphate can be neutralized^54^ or other organic matter having hydrophobic adsorption to the CeNP surfaces may lead to a higher affinity between the particles. Indeed, Mosley and Hunter^55^ reported that adsorption of organic matter suppresses aggregation through the formation of steric barriers, whereas it can also prevent dissociation of colloid aggregate once they formed aggregates. Because of such effects owing to the presence of other small organic matters in ES, the ES + CeNPs case was found to be slightly less aggregated than that of the PS + 160 μM P + CeNPs case. Figure S4 shows the results of additional experiments measuring the sedimentation rates of CeNPs at pHs of 2.0, 3.0, 3.5, 6.0, 7.0, and 10.0 under three conditions: CeNPs, 160 μM P + CeNPs, and ES + CeNPs. These rates were calculated based on the turbidity time course monitored using UV-Vis spectroscopy. The sedimentation rate of CsNPs was the fastest when the solution pH is close to the iep: 160 μM P + CeNPs at pH 2, ES + CeNPs at pH 3.5, and CeNP at pH 7.0. The sedimentation rate of CeNPs greatly depends on the solution pH, which is consistent with the DLS analysis.

### Comparison with the other macromolecules

**Figure 8:**
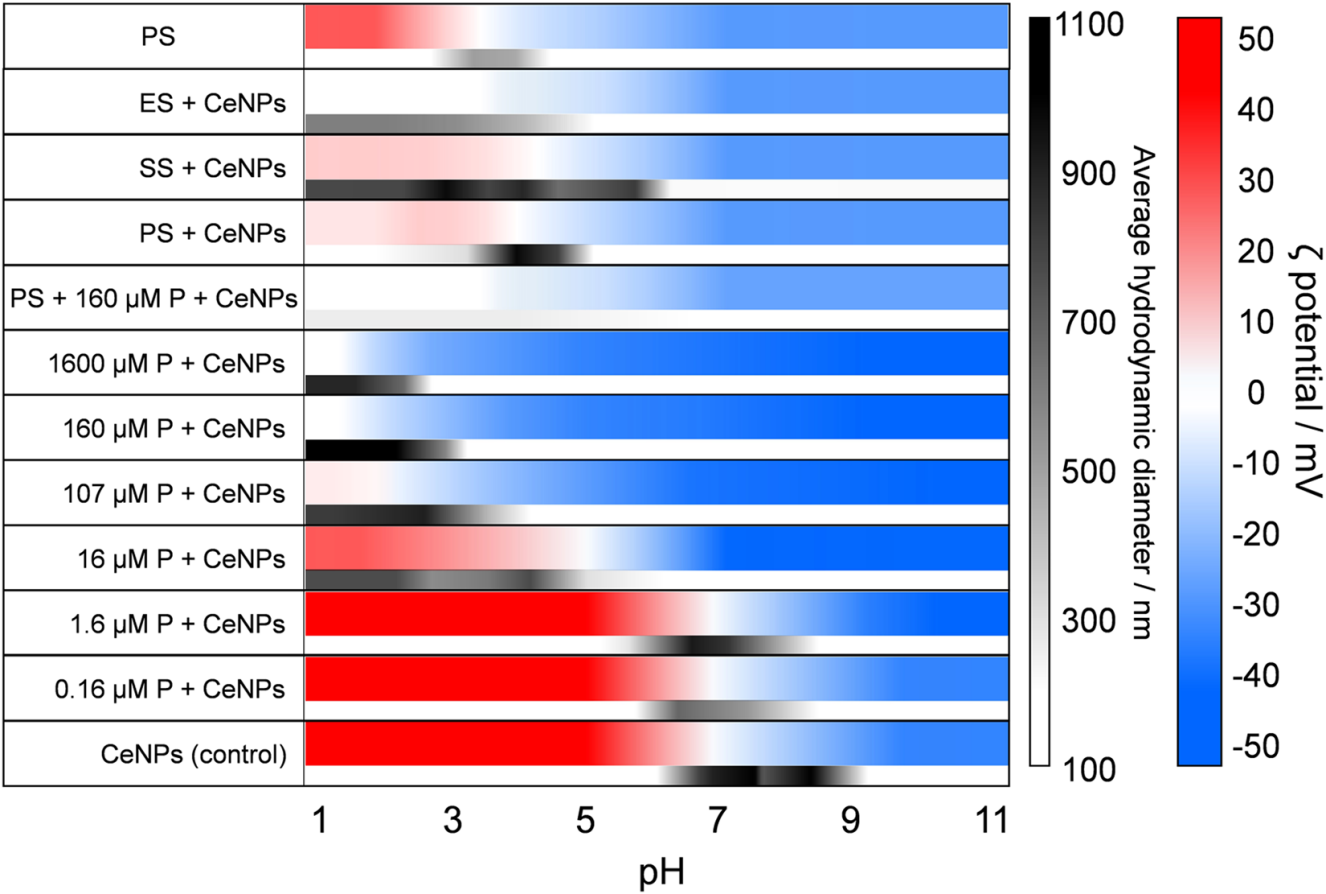
Size and zeta potential of the CeNP aggregates or ES in the present experimental conditions. The blue–red color scale represent the zeta potential while the black and white scale indicates the average hydrodynamic diameter.

Figure 8 summarizes the surface properties and sizes of CeNPs aggregates under the present experimental conditions. Because the ES is a part of natural organic matter (NOM), and vice versa, it is useful to compare the present results with the well-known effects of NOM such as humic substances (HS) on the aggregation behavior of various engineered nanoparticles^19,23,25,56–61^. The presence of NOM typically stabilizes the colloids and reduces aggregation by coating the nanoparticle surfaces: forming a steric barrier and modifying the surface charges^62^. Our results on the role of microbial ES appear to be similar to that of NOMs previously reported^63^. The similarity between the effects of exudate and NOM on the aggregation behavior of TiO_2_ nanoparticles has already been recognized^64^. However, comparing the elemental analysis and FTIR data of ES (Table 1) with those of fulvic acid (FA) and humic acid (HA)^65^, the ES contains higher concentrations of nitrogen, reflecting a higher protein and polypeptides content, whereas HS consist of carbohydrates, alginate, amino acids, lignin, and pectin. The clear difference between ES and NOM is the preferential adsorption of protein and polypeptides to the CeNP surfaces.

In general, the molecular weights of the differently sized components in HS, FA and HA, range from a few hundred to thousands and from thousands to several millions of Da, respectively^65^. Several studies have reported that large molecular size of HA can reduce aggregation more efficiently than FA^66,67^ by forming thick polymer layers. In the case of NOM, it has been reported that adsorbed layer thickness, aromaticity, and molecular weight, are all correlated with aggregation behavior^26,63^. It is also noted that typical surfactants with low-molecular weights can desorb or be displaced by larger molecules, such as NOMs^63^; however, such phenomena were not observed in the present experiments. This can be ascribed to the fact that the functional groups of the organic substances that adsorbed onto the CeNP surfaces were similar in SS and PS. This is reasonable because proteins and polypeptides, which preferentially adsorbed to the CeNP surfaces in the present experiment, generally adsorb by forming chemical bonds^37,45^.

Furthermore, in case of NOMs, large fibrillary polymers can form bridges between nanoparticles, and this occasionally promotes aggregation^68^. Such an enhanced aggregation mechanism did not occur in our PS case because the conformation of the proteins and polypeptides present, as revealed in the AFM images (Fig. 2), were approximately spherical. Rather, the PS further reduced aggregation compared to the SS case (Fig. 8).

## Conclusion

Adsorption experiments using ES (PS + SS), PS (>1 kDa), and SS (<1 kDa) to CeNPs were conducted to understand the effect of each fractionated component on the modification of the surface and dispersibility of the CeNPs at pHs ranging from 1.5 to 10. Microscopic and spectroscopic characterization of these components, with and without CeNPs, revealed that the three fractions were composed of organic matter that contained similar functional groups, despite the size difference of the molecules within each fraction because polypeptides and amino acids were present in the SS fraction. The preferential adsorption of proteins and/or polypeptides, and inorganic phosphates were observed in all fractions. The polymeric substances in the ES case formed aggregates as large as a few tens of nm and the chemical composition showed heterogeneous distributions on the CeNP surfaces from the adsorption of multiple components. The ζ potential of CeNP with ES, PS, and SS exhibited the same pH dependence, suggesting that the polymeric substances, even smaller-sized molecules, modified the surface charge of the CeNPs with apparent similarity to the PS case. Although the ζ potentials were governed by the polymeric substances in the ES, other small polymers with different ieps can also adsorb to the CeNP surfaces and reduce nanoparticle aggregation under conditions where the ζ potential is nearly zero. Thus, the suppression effects on the aggregation by ES adsorption onto CeNPs can be expressed under wider pH conditions than those derived from PS adsorption only.

There is a wide range of amounts and chemical variations in small and large polymeric substances, as well as geochemical parameters, in natural surface and subsurface environments. Hence, the results of the present study highlight the non-negligible impact of microbially derived polymeric substances, of various molecular sizes, on the migration of CeNP in the environment. The dynamics of adsorption, aggregation, and transformation of various organic matter–nanoparticle couples in realistic environments still remains to be explored as previously pointed out^69^. The quantitative data obtained in the present study can be useful for understanding the role of small-sized molecules derived from microbial activity on the migration of CeNPs in aquatic environments, especially where bacterial consortia prevail.

## Electronic supplementary material

Supplementary Information
