# Supplementary material for: The competing effects of microbially derived polymeric and low molecular-weight substances on the dispersibility of CeO2 nanoparticles
Source: Sci Rep. 2018 Feb 26;8:3648. doi: 10.1038/s41598-018-21976-9 (PMC5827655; doi:10.1038/s41598-018-21976-9)
Supplement: Supplementary file 1 — Supplementary Information [file 41598_2018_21976_MOESM1_ESM.pdf]

Supporting material for:

The competing effects of microbially derived polymeric and low molecular-weight substances on the dispersibility of CeO_2 nanoparticles.

By Yuriko Nakano, Asumi Ochiai, Keisuke Kawamoto, Ayaka Takeda, Kenta Ichiyoshi, Toshihiko Ohnuki, Michael F. Hochella, Jr.

and Satoshi Utsunomiya

Methods for aggregation and sedimentation experiments

Prior to the sedimentation experiments, the stock suspension of CeNPs was sonicated for 10 min. Aliquots of the CeNPs solution were added to three types of solutions: (i) 1 mM NaCl solution (control); (ii) 1 mM NaCl + 160 μM H_3PO_4 solution (160 μM P); and (iii) ES solution that contained 1 mM NaCl solution. The CeNP concentration was set at 100 mg L^{-1} and the pH was adjusted to 2.0, 3.0, 3.5, 6.0, 7.0, or 10.0. The dynamic aggregation process was monitored using a UV-vis spectrophotometer (V-530; Jasco, Tokyo, Japan) by measuring the back-scattering intensity of the CeNPs at 340 nm as a function of time. The absorbance was measured every 30 s for 150 min.

Table S1. The concentrations of dissolved organic carbon before and after the adsorption experiments

sample	Before adsorption (ppm)	After adsorption (ppm)
ES	171.7	163.0
PS	23.3	18.4
PS + P	23.3	20.6
SS	36.1	33.5

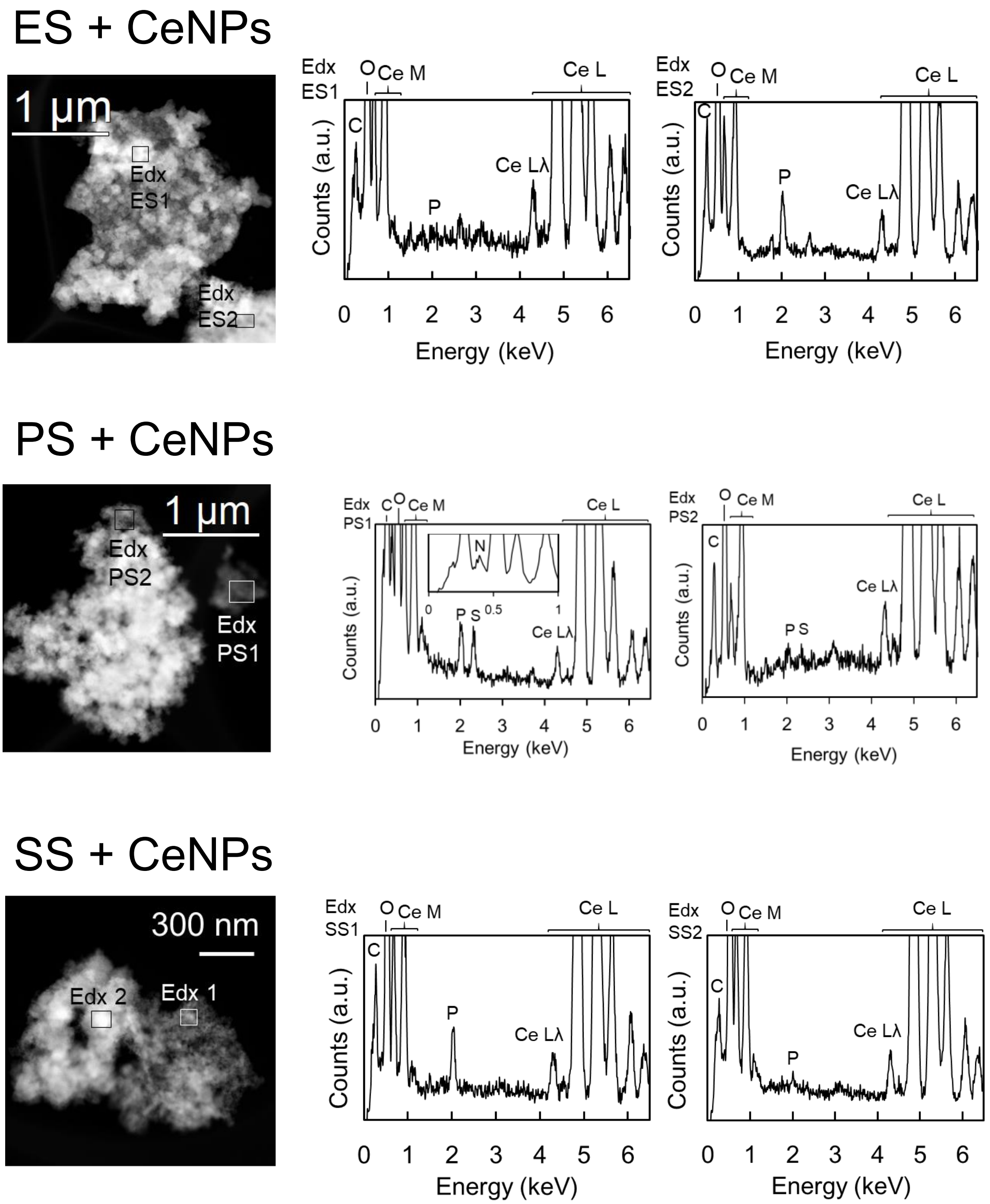

Fig. S1. Representative EDX spectra of ES + CeNPs, PS + CeNPs, and SS + CeNPs.

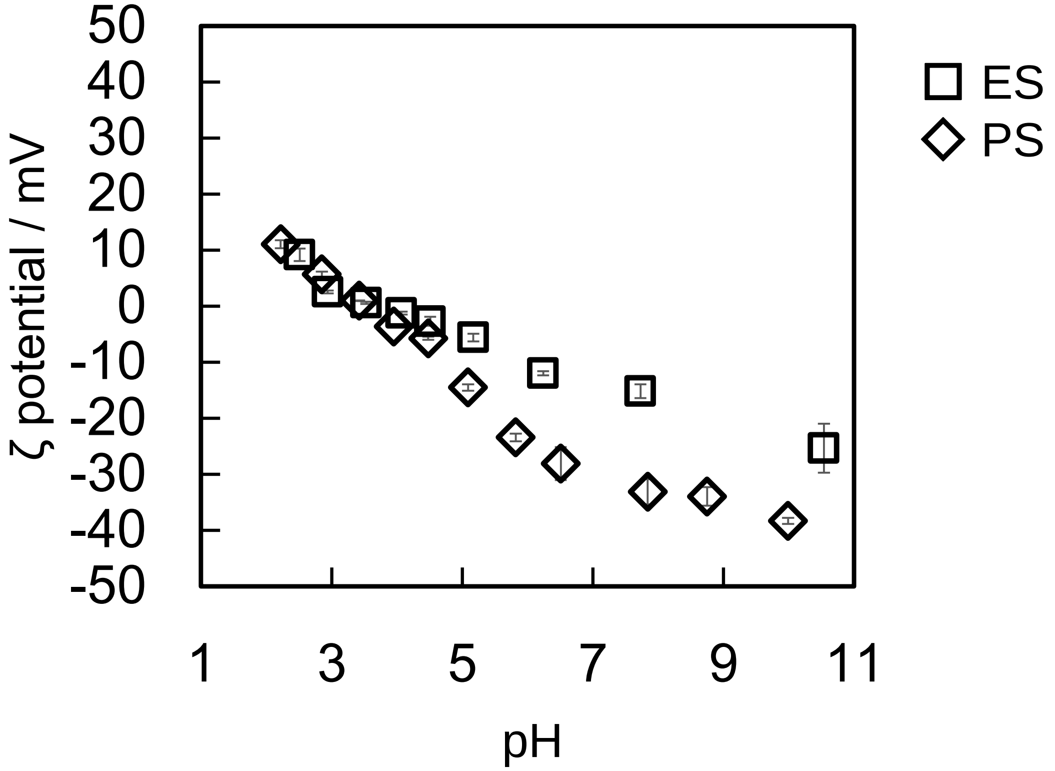

Fig. S2. The *ζ* potential of ES in comparison with that of PS.

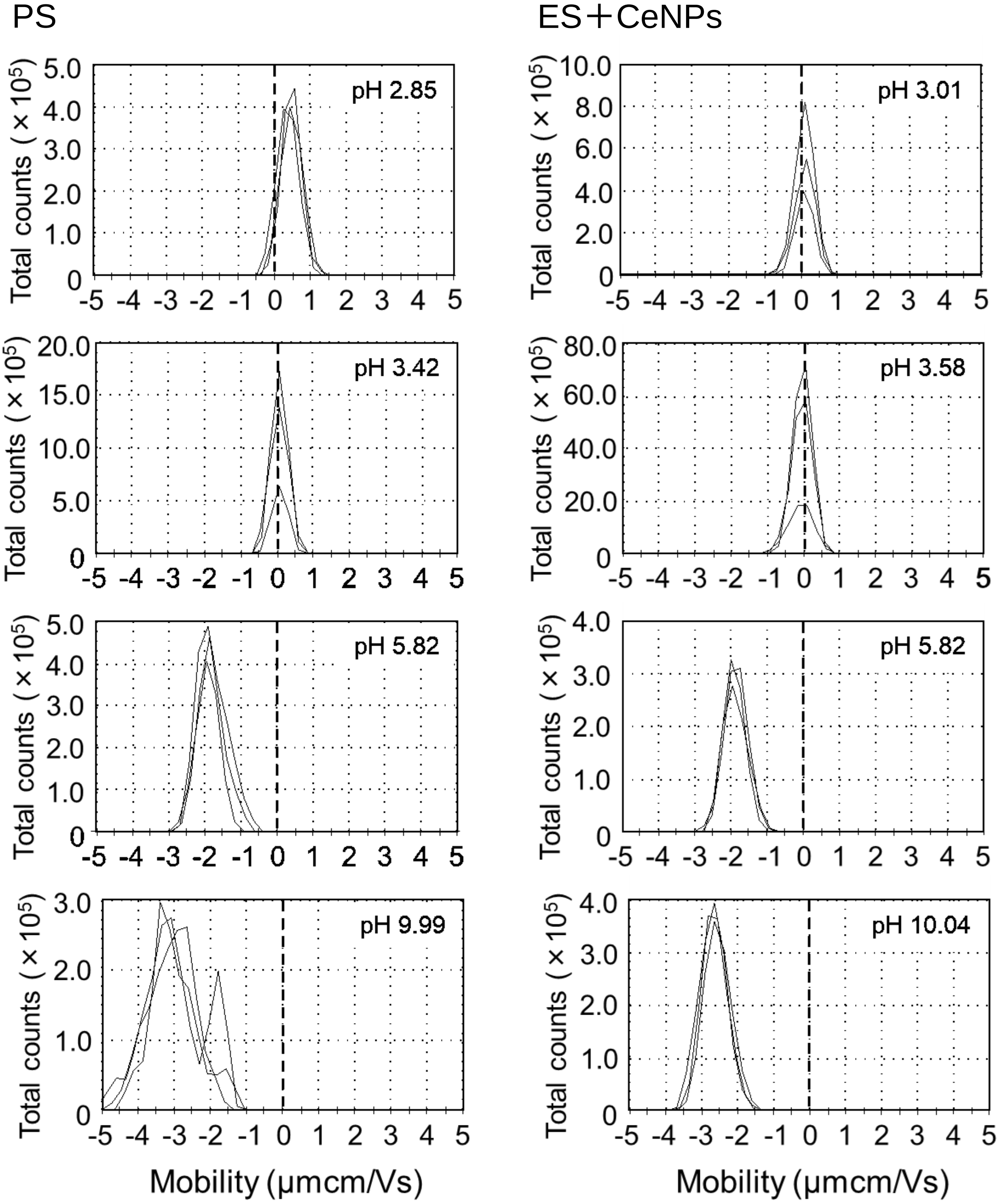

Fig. S3. Electrophoretic mobility distribution of PS (left panels) and ES + CeNPs (right panels) measured during the ζ potential measurement for Fig. 5a.

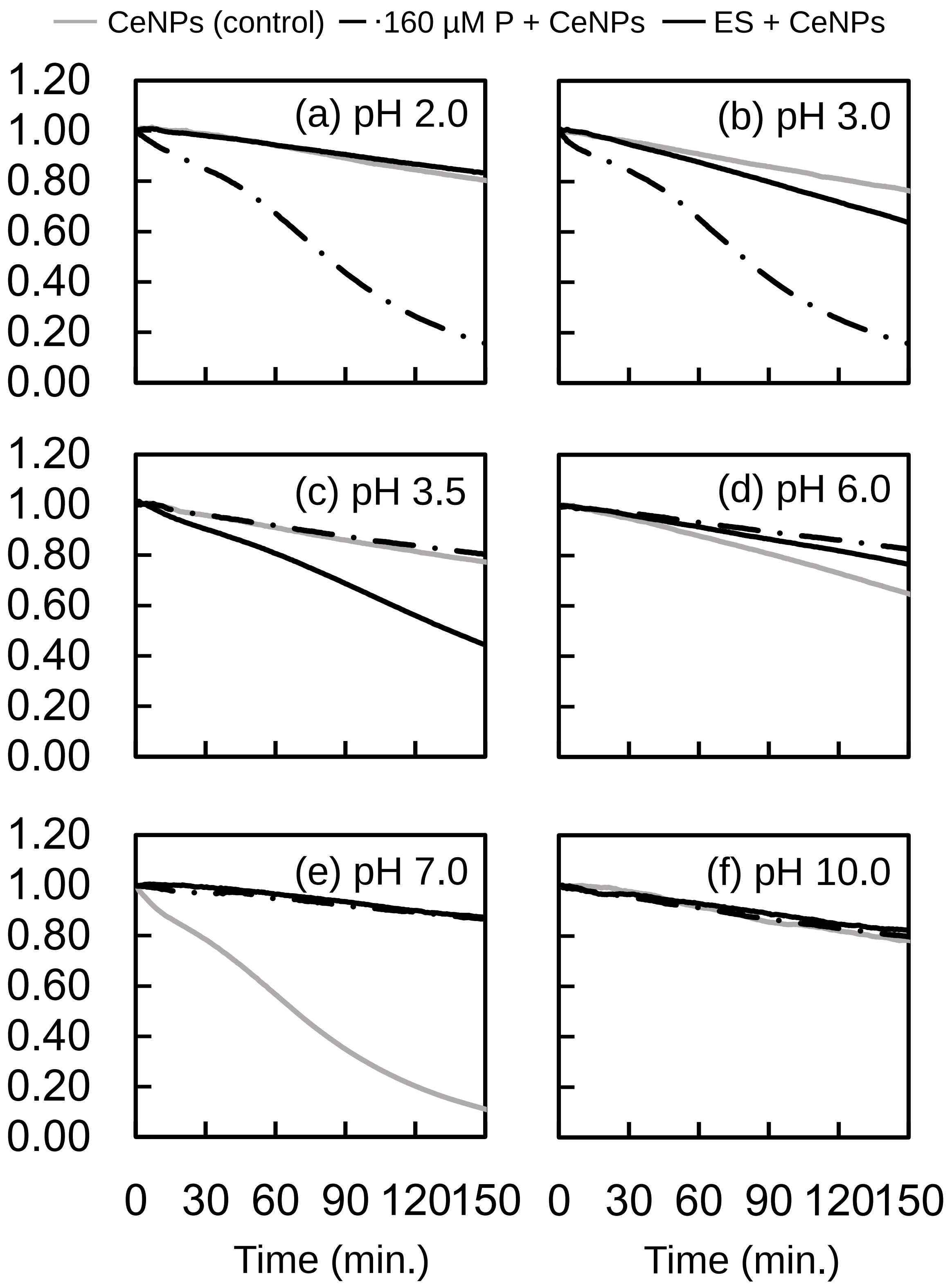

Fig. S4. Time-course of turbidity during the settling experiment of CeNPs in 1 mM NaCl for 1–150 min under various conditions: (a) pH of 2.0, (b) 3.0, (c) 3.5, (d) 6.0, (e) 7.0, and (f) 10.0. Gray line: CeNPs (control), dashed line: 160 μM P + CeNPs, black line: ES + CeNPs.
